# Dscam1 Has Diverse Neuron Type-Specific Functions in the Developing *Drosophila* CNS

**DOI:** 10.1523/ENEURO.0255-22.2022

**Published:** 2022-08-26

**Authors:** Nicole Wilhelm, Shikha Kumari, Niklas Krick, Christof Rickert, Carsten Duch

**Affiliations:** Institute for Developmental Biology and Neurobiology, Johannes Gutenberg University Mainz, 55128 Mainz, Germany

**Keywords:** dendrite, *Drosophila*, Dscam1, interneuron, motorneuron, self-avoidance

## Abstract

Two key features endow *Drosophila* Down syndrome cell adhesion molecule 1 (Dscam1) with the potential to provide a ubiquitous code for neuronal arbor self-avoidance. First, Dscam1 contains three large cassettes of alternative exons, so that stochastic alternative splicing yields 19,008 Dscam1 isoforms with different Ig ectodomains. Second, each neuron expresses a different subset of Dscam1 isoforms, and isoform-specific homophilic binding causes repulsion. This results in even spacing of self-arbors, while processes of other neurons can intermingle and share the same synaptic partners. In principle, this Dscam1 code could ensure arbor spacing of all neurons in *Drosophila*. This model is strongly supported by studies on dendrite spacing in the peripheral nervous system and studies on axonal branch segregation during brain development. However, the situation is less clear for central neuron dendrites, the major substrate for synaptic input in the CNS. We systematically tested the role of Dscam1 for dendrite growth and spacing in eight different types of identified central neurons. Knockdown of Dscam1 causes severe dendritic clumping and length reductions in efferent glutamatergic and aminergic neurons. The primary cause for these dendritic phenotypes could be impaired self-avoidance, a growth defect, or both. In peptidergic efferent neurons, many central arbors are not formed, arguing for a growth defect. By contrast, knockdown of Dscam1 does not affect dendrite growth or spacing in any of the five different types of interneurons tested. Axon arbor patterning is not affected in any neuron type tested. We conclude that Dscam1 mediates diverse, neuron type-specific functions during central neuron arbor differentiation.

## Significance Statement

Seminal studies have demonstrated that alternative splicing of *Drosophila* Down syndrome cell adhesion molecule 1 (Dscam1) generates thousands of cell surface proteins with isoform-specific binding. Each neuron produces different subsets of Dscam1 isoforms, so that homophilic interactions can provide a code for self-recognition and self-avoidance. This is the confirmed mechanism for dendrite spacing of mechanosensory neurons in the PNS and for axonal branch segregation during mushroom body development. Therefore, Dscam1 isoform diversity has become a prime example for a probabilistic code precisely regulating brain development. This study shows that Dscam1 is not required for dendrite spacing in numerous types of central neurons and exhibits differential effects on dendrite growth, thus refining the current concept of Dscam1 function during brain development.

## Introduction

Dendrite morphology is essential for the correct wiring of neural circuitry (Libersat and Duch, 2004; Lefebvre et al., 2015), for the integration of synaptic input (London and Häusser, 2005; Stuart and Spruston, 2015), and, thus, for correct brain development and function. One prominent dendritic morphology feature displayed by most types of neurons is even spacing of branches (Gao, 2007), an architecture trait for complete but nonredundant coverage of the input space of a neuron by its dendritic branches (Hattori et al., 2008). A possible mechanism for even dendrite spacing is self-avoidance, the tendency of dendritic branches of the same neuron to avoid each other but ignore branches of other neurons (Zipursky and Grueber, 2013). Selective self-avoidance requires a means for self-recognition. In *Drosophila melanogaster*, seminal studies have demonstrated that alternative splicing of Down syndrome cell adhesion molecule 1 (Dscam1) can provide a nervous system-wide code for self-avoidance (Zipursky and Grueber, 2013; Kise and Schmucker, 2013). Here, we test systematically whether this Dscam1 isoform code is used ubiquitously throughout the CNS to ensure even dendrite spacing in different types of neurons.

Dscam1 is a member of the Ig superfamily and encodes cell surface receptors with an intracellular C terminus, a transmembrane domain, and an ectodomain with 10 Ig domains and 6 fibronectin repeats. Probabilistic splicing of alternative exons results in ∼19,000 isoforms that differ in 1–3 of the 10 Ig domains (Schmucker et al., 2000). One additional alternative exon in the transmembrane domain controls axonal versus dendritic localization of Dscam1 protein (Wang et al., 2004; Shi et al., 2007; Yang et al., 2008). Importantly, only the same ectodomains interact with each other (Wojtowicz et al., 2004, 2007; Meijers et al., 2007; Sawaya et al., 2008; Wu et al., 2012) to mediate homophilic repulsion, and each neuron expresses a unique subset of ∼5–50 Dscam1 ectodomain isoforms (Neves et al., 2004; Zhan et al., 2004). Consequently, probabilistic splicing suffices to make the Dscam1 isoform composition of each neuron different from that of its neighbors. This provides each neuron in the nervous system with a unique code for self-avoidance. In fact, numerous studies have provided compelling evidence that the Dscam1 code is required for correct dendrite spacing in sensory neurons (Hughes et al., 2007; Matthews et al., 2007; Soba et al., 2007) and antennal lobe neurons (Zhu et al., 2006), as well as for the formation of axon bifurcations in mechanosensory neurons (He et al., 2014) and the segregation of axonal sister branches during mushroom body (MB) development in the central brain (Wang et al., 2002, 2004; Zhan et al., 2004; Hattori et al., 2007, 2009). An attractive hypothesis is that Dscam1 may be used as a nervous system-wide mechanism for self-avoidance in all *Drosophila* neurons with branched axons or dendrites (Kise and Schmucker, 2013).

However, in adult *Drosophila* flight motoneurons (MNs) that differentiate during metamorphosis (Consoulas et al., 2002), Dscam1 is required for dendrite growth rather than dendritic self-repulsion, although it remains unclear whether the primary cause for reduced dendrite growth is impaired filopodial self-avoidance during growth-cone sprouting (Hutchinson et al., 2014; Ryglewski et al., 2014b). Moreover, it has been suggested that MB axonal wiring may depend on influences of Dscam1 isoform bias with nonrepulsive signaling (Hong et al., 2021). Here we test for the generality of Dscam1-mediated self-avoidance during *Drosophila* CNS differentiation. Although Dscam1 is abundantly expressed in all central neuropils at critical stages of neuronal differentiation, our data suggest that the Dscam1 code is not used for arbor spacing by all central neuron types. Instead, Dscam1 is required for normal dendrite growth in multiple different types of efferent neurons. In octopaminergic and glutamatergic efferent neurons, the primary cause could be impaired filopodial self-avoidance, whereas in crustacean cardioactive peptide (CCAP)-releasing peptidergic neurons many arbors are not formed, indicating a primary growth defect. Finally, knockdown (kd) of Dscam1 has no effect on dendrite differentiation in any of the five different types of interneurons tested. We conclude that Dscam1 has diverse, cell type-specific effects on neuronal arbor differentiation.

## Materials and Methods

### Animals

Fruit flies, *D. melanogaster*, were reared on a standard yeast-cornmeal-agar-glucose diet [as follows (g/L H_2_O): 121 glucose, 57 cornmeal, 11 agar, 30 active dry yeast, 0.6 ascorbic acid) at 25°C and 60% humidity under a 12 h light/dark regime. For experiments, third instar larva, different pupal stages, and adult flies (1–4 d after adult emergence) of both sexes were used. Staging of pupae was performed by external anatomic criteria (Bainbridge and Bownes, 1981), where the 90–100 h of *Drosophila* pupal life are divided into 15 stages with varying duration.

For visualization of Dscam1 spatiotemporal expression patterns through the *Drosophila* CNS during postembryonic life and for assessing knock-down efficacy by Western blotting, we used an EGFP-tagged Dscam1 fly strain (w; Dscam1^MiMICGFP^) that was generated by Kamiyama et al. (2015; Table 1). Fly stocks to generate homozygous Dscam1 mutant larval motoneurons in a heterozygous control background (MARCM) were as follows: hsFLP, C155-Gal4, UAS-mCD8-GFP; FRT42D, and tubP-GAL80/CyO (Matthews et al., 2007) crossed to FRT42D Dscam21/CyO (Hummel et al., 2003), FRT42D Dscam23/CyO, or FRT42D Dscam47/CyO (Matthews et al., 2007). Dscam1 mutant fly strains were a gift from Wesley Gruber (Columbia University, New York, NY).

**Table 1:** Fly strains used in this study with sources and research resource identifiers

Fly strains used	Abbreviation	Source	Figures	RRID
w; Dscam1^M^*^i^*^MICGFP^	Dscam1 protein trap	Kamiyama et al. (2015)	1, 2	
P{w[+mW. h]=GawB}elav[C155]	Elav-GAL4	BDSC 458	2	BDSC_458
w^1118^; P{GD14362}v36233	Dscam1-RNAi	VDRC 36233	2, 3, 4	FlyBase_FBst0461591
w^1118^; P{KK100296}VIE-260B	Dscam1-RNAi	VDRC 108835	2, 3, 4	FlyBase_FBst0482167
w[1118]; P{w[+mC]=UAS-Dcr-2.D}2	UAS-dcr2	BDSC 24650	2, 3, 4	BDSC_24650
w[1118]; P{w[+mC]=UAS-Dcr-2.D}10	UAS-dcr2	BDSC 24651	2–7	BDSC_24651
y^1^ w^67^*^c^*^23^; P{GawB}Hr39*^c^*^739^	C739-GAL4	BDSC 7362	2	BDSC_7362
w^1118^	W1118	BDSC 3605	2, 3	BDSC_3605
w[*]; P{w[+mC]=Tdc2-GAL4.C}2	TDC2-GAL4	BDSC 9313	5	BDSC_9313
y1 w*; P{CCAP-GAL4.P}16/CyO	CCAP-GAL4	BDSC 25685	5	BDSC_25685
y1 w*; P{GAL4-per.BS}3	Per-GAL4	BDSC 7127	6	BDSC_7127
w[1118]; P{y[+t7.7] w[+mC]=GMR20B01-GAL4} attP2	Basin-1-GAL4	BDSC 48877	6	BDSC_48877
w[1118]; P{y[+t7.7] w[+mC]=GMR60F02-GAL4} attP2	CSD-GAL4	BDSC 48228	7	BDSC_48228
w[1118]; P{y[+t7.7] w[+mC]=R47H03-p65.AD} attP40; P{y[+t7.7] w[+mC]=R72E01-GAL4. DBD}attP2/TM6B, Tb[1]	Split-GAL4 for LC4	BDSC 68259 (Wu et al., 2016)	7	BDSC_68259
w[1118];P{R17A04-p65.AD}attP40 P{UAS-CD4:: tdGFP}7M1/Plum-Balancer;P{R68A06-GAL4. DBD}attP2/TM6b Tb1	Split-GAL4 for GF	BDSC 79602 (von Reyn et al., 2014)	7	BDSC_79602
yw; UAS-Dscam1.30.30.2	UAS-Dscam1	Zhan et al., 2004	7	
hsFLP, C155-Gal4, UAS-mCD8-GFP; FRT42D, tubP-GAL80/CyO	MARCM ready	Matthews et al., 2007	3	
FRT42D Dscam21/CyO	Dscam21 MARCM ready	Hummel et al., 2003	3	
FRT42D Dscam23/CyO	Dscam23 MARCM ready	Matthews et al., 2007	3	
FRT42D Dscam47/CyO	Dscam47 MARCM ready	Matthews et al., 2007	3	

RRID, Research resource identifiers; BDSC, Blooming Drosophila Stock Center.

We used the binary GAL4/UAS expression system to target transgenes to selected types of neurons. For targeted overexpression of UAS-Dscam1 under the control of GAL4, we used a Dscam1 isoform that contains the alternative exons 4.1, 6.30, 9.30, and 17.2 (Zhan et al., 2004). For targeted RNAi kd of Dscam1 (Dscam1-kd), we used different UAS-RNAi transgenes [catalog #36233 and #108835, Vienna Drosophila Resource Center (VDRC)]. In all RNAi experiments, inclusion of extra Dicer-2 (UAS-dcr2; catalog #24650, Bloomington Drosophila Stock Center; Dietzl et al., 2007) was used to increase knock-down strength, as previously reported (Ryglewski et al., 2014b; Hutchinson et al., 2014). In all experiments with UAS-Dscam1-RNAi and UAS-Dcr2 driven by GAL4, as control we used UAS-Dcr2 alone driven with in same GAL4 fly strain.

To restrict UAS-Dscam1-RNAi (catalog #36233 or #108835, VDRC) expression to two identified MNs per larval ventral nerve cord hemisegment, GAL4 was expressed in MN1-Ib and in MNISN-1 under the control of the even-skipped promoter (RN2-GAL4; catalog #7473, Bloomington Drosophila Stock Center; Fujioka et al., 2003). MN1-1b is also named aCC by its embryonic location (Fujioka et al., 2003), has axon terminals with big type I boutons and innervates muscle 1. MNISN-Is is also named RP2, innervates multiple dorsal muscles via the intersegmental nerve (ISN) and has small type I terminals (Hoang and Chiba, 2001). A flippase strategy was used to express UAS-RNAi transgenes in a mosaic fashion under the control of the strong actin-GAL4 promoter. The flippase is driven by the weak RN2 promoter to remove a stop cassette to activate the strong actin-GAL4 promoter. This resulted in strong RNAi expression in only a small subset of aCC and RP neurons (Kadas et al., 2017). This scheme produces genetic mosaics, in which non-GFP-labeled MNs serve as internal controls.

The following GAL4 driver strains were used to restrict UAS-Dscam1-RNAi; UAS-dcr2, and UAS-GFP or UAS-tdTomato to specific types of neurons. C739-GAL4 (catalog #7326, Bloomington Drosophila Stock Center) was used to target expression to adult MB; TDC2-GAL4 for tyrosine decarboxylase 2-expressing octopaminergic neurons (Cole et al., 2005; Schützler et al., 2019); CCAP-GAL4 (catalog #25658, Bloomington Drosophila Stock Center) for crustacean cardioactive peptide-expressing neurons in the larval and the adult ventral nerve cord (VNC), per-GAL4 (catalog #7127, Bloomington Drosophila Stock Center) for period-expressing interneurons in the larval VNC; 60F02-GAL4 (catalog #48228, Bloomington Drosophila Stock Center) for serotonergic interneurons in the adult brain; R20B01-GAL4 (catalog #48877, Bloomington Drosophila Stock Center) for basin-1 interneurons in the larval VNC; a split-GAL4 strain (catalog #68259, Bloomington Drosophila Stock Center) for lobular columnar type 4 (LC4) visual interneurons (Wu et al., 2016); and a split-GAL4 strain (catalog #79602, Bloomington Drosophila Stock Center) for the giant fiber (GF) interneurons (von Reyn et al., 2014).

### Western blotting

Dscam1-kd efficacy was assessed by Western blotting with larval CNS homogenate from w; Dscam1^MiMICGFP^ flies. Animals were stunned on ice for 10 min and dissected in ice-cold saline. Ten CNSs were collected in 30 μl of ice-cold 2× SDS sample buffer with dithiothreitol (DTT; 25 ml of 4× Tris CI/SDS, pH 6.8, 20 ml of glycerol, 4 g of SDS, 0.31 g of DTT, and 1 mg of Bromophenol blue, added to 100 ml of double-distilled H_2_O (ddH_2_O)]. Samples were homogenized and boiled at 96°C for 3 min and then stored at − 28°C. A 5% (bis-acrylamide) stacking gel [6.8 ml of ddH_2_O, 1.7 ml of 30% bis-acrylamide, 1.25 ml of 4× Tris/SDS, pH 6.8, 100 μl of 10% ammonium persulfate, and 10 μl of tetramethylethylenediamine (TEMED)] and a 5% (bis-acrylamide) running gel (18.6 ml of ddH_2_O, 10.7 ml of 30% bis-acrylamide, 10 ml of 4× Tris/SDS, pH 8.8, 400 μl of 10% ammonium persulfate, and 16 μl of TEMED) was used. After gel pouring, the pockets were washed with SDS-glycine-Tris electrophoresis buffer (3 g of Tris base, 14.4 g of glycine, 1 g of SDS, added to 200 ml of ddH_2_O). Before loading, the samples were boiled at 96°C and centrifuged at 10,000 × *g* for 1 min. The gel was run at 0.02 A until the dye front passed the stacking gel, then the current was decreased to 0.01 A (PowerPac, BIO-RAD). Proteins were blotted onto nitrocellulose at 4°C overnight at 40 V (PowerPac, BIO-RAD) in a wet tank filled with transfer buffer (18.2 g of Tris base, 86.5 g of glycine, and 900 ml of methanol, and adding ddH_2_O to 6 L). After blotting, the membrane was cut in half at ∼140 kDa (between the size of the Dscam1^GFP^ band and the HSP90 loading control band). Next, membranes were washed with ddH_2_O for 10 min, incubated with TBST (10 ml of 1 m Tris, pH 7.5, 30 ml of 5 m NaCl, and 1 ml of Tween 20, added to 1000 ml with ddH_2_O) three times for 20 min, and blocked with 10% dried milk-TBST solution for 2 h. Next, membranes were incubated separately with primary antibody [rabbit anti-GFP: 1:1000; catalog #A-11122, Thermo Fisher Scientific (RRID:AB_221569); and rabbit-anti-hsp90: 1:1000; catalog #4874S, Cell Signaling Technology (RRID:AB_2121214)] diluted in 2.5% milk-TBST solution at 4°C overnight. Membranes were then washed with TBST three times for 20 min before incubation with secondary antibody [goat anti-rabbit IgG: 1:10,000; catalog #111–035-144, Jackson ImmunoResearch (RRID:AB_2307391)] in TBST for 2 h at 25°C. After washing three times for 20 min with TBST and 20 min with TBS, membrane was incubated in Immobilon Western Chemiluminescent HRP substrate (catalog #WBKLS0500, Millipore) for 3 min. Detection was conducted with a Fusion SL Camera and Fusion software (Vilber Lourmat).

### Immunocytochemistry

Immunolabeling was conducted to assess the structure of GFP or tdTomato-expressing neurons with and without genetic manipulation of Dscam1. Dissection of larval, pupal, and adult *Drosophila* was conducted in HL3.1 saline containing the following (in mm): NaCl 70, KCl 2.5, MgCl_2_ 2, CaCl_2_ 0.5 or 2, NaHCO3 10, trehalose 5, sucrose 115 or 109 (when CaCl_2_ was 2), and HEPES 5, with pH adjusted to 7.24–7.25 with 1N NaOH, at 310 mOsm, as previously described (third instar larvae, Kadas et al., 2017; pupae, Ryglewski et al., 2014a; adult, Ryglewski and Duch, 2009). Following dissection, specimens were fixed in 4% paraformaldehyde (in 0.1 m PBS) for 45 min, then washed in PBS (4× 30 min) and in PBS-Triton X-100 (0.5% in 0.1 m PBS) 6× for 20 min at room temperature. Next, specimens were incubated shaking with primary antibodies [rabbit anti-GFP: 1:1000; Cat# A-11122, Thermo Fisher Scientific (RRID:AB_221569); or rat-anti-mCherry: 1:5000, monoclonal antibody 16D7, catalog # M11217, Thermo Fisher (RRID:AB_2536611)] in 0.3% PBS-Triton X-100 at 4°C overnight. This was followed by washing 8× 30 min in PBS at room temperature and incubation with secondary antibodies (donkey anti-rabbit Alexa Fluor 488: 1:500; catalog #711–545-152, Jackson ImmunoResearch (RRID:AB_2313584); or donkey anti-rat Alexa Fluor 594: 1:500; catalog #712–587-003, Jackson ImmunoResearch (RRID:AB_2340692)] at 4°C for 12 h. Finally, specimen were washed 6× 30 min in PBS, dehydrated in an ascending EtOH series (50, 70, 90, and 100%, for 10 min each), and cleared and mounted in methylsalicylate. Triple labeling of neuromuscular axon terminals with anti-HRP for neuronal membrane, anti-GFP to report GAL4-positive neurons, and anti-Bruchpilot (brp) antibodies for presynaptic active zones (AZs) was conducted as described in the study by Krick et al. (2021).

### Confocal laser-scanning microscopy

All images were acquired with a confocal laser-scanning microscopy (CLSM; model SP8, Leica) with either a 20× or a 40× oil-immersion lens at resolution of 1024 × 1024 pixels and 8 bit gray depth. Alexa Fluor 488 was excited with an argon laser at 488 nm, and emission was detected with a photomultiplier wavelength between 495 and 530 nm. Alexa Fluor 594 was excited with a 561 nm DPSS (diode-pumped solid-state) laser and detected with a gallium arsenide hybrid detector between 580 and 620 nm. For high-resolution imaging with a 40× oil lens (numerical aperture 1.3), *z*-step size was 0.3 μm, and the zoom factor was up to 3.5. The resulting voxel dimensions were 86 × 86 × 290 nm (*x*, *y*, *z*). In some cases, dendritic structure was reconstructed from confocal image stacks after export to Amira software (AMIRA 4.1.1; FEI) with custom plug-ins (Schmitt et al., 2004; Evers et al., 2005).

### Two-electrode voltage-clamp recordings of larval muscle

For all two-electrode voltage-clamp recordings, dissected third instar larvae were mounted under a 20× water-immersion lens on an upright microscope (model BX51WI, Olympus) and continuously perfused with HL3.1 saline (see above). M6/7 or M4 muscle was impaled with two thin-walled borosilicate glass electrodes with filament (outer diameter, 1 mm; inner diameter, 0.5 mm; Sutter Instrument), which were pulled with a P-97 Flaming/Brown Electrode Puller (Sutter Instrument), and had tip resistances of 8–10 MΩ for current injection and 20–25 MΩ for voltage recording. Electrodes were filled with 3 m KCl, and recordings were acquired with an AxoClamp 2B amplifier connected to a Digidata 1550 digitizer, and controlled by PCamp10.7 software (all from Molecular Devices Molecular Devices) at a sampling rate of 50 kHz with a 25 kHz anti-alias filtering.

For quality control, only recordings with an initial muscle membrane potential below −50 mV were accepted. The motor nerve to M6/7 or M4, respectively, was pulled into a custom-made suction electrode. Motoneuron action potentials were evoked by extracellular voltage stimulation through the suction electrode of 2–4 V amplitude and 0.1 ms duration with an isolated pulse stimulator (model 2100, A-M Systems). Spontaneously occurring and evoked postsynaptic currents were recorded at a holding potential of −70 mV.

### Quantification of active zone numbers and bouton sizes

The brp dots, as obtained by immunolabeling and CLSM microscopy, are commonly used as AZ markers (Krick et al., 2021). For the counting of brp dots, we made use of the scikit-image library for Python (van der Walt et al., 2014). We normalized intensities of the brb channel stacks with an inbuilt scikit-image function (skimage.exposure.rescale_intensity). Then we used a Laplacian of Gaussian algorithm (skimage.feature.blob_log) that reliably detects bright image spots on darker backgrounds. We kept the default parameters of the blob_log function, but only changed max_σ to 3, num_σ to 5, and threshold to 0.07.

### Experimental design and statistical analysis

**Figure 1.**
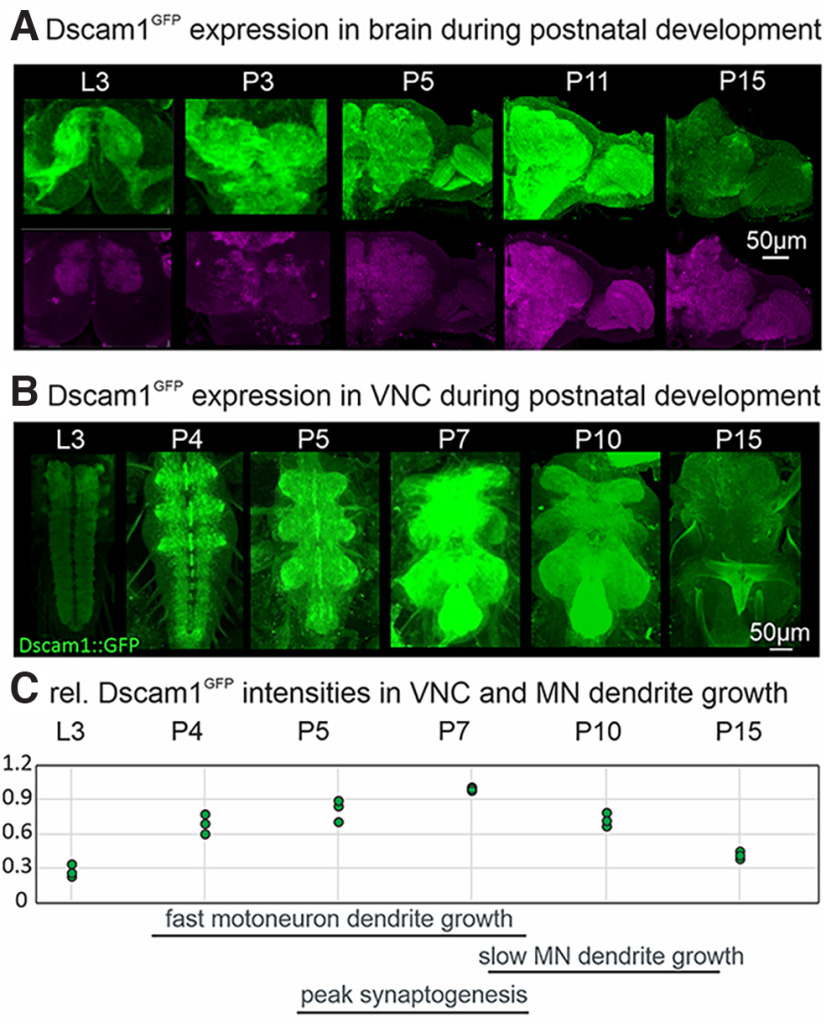
Temporal and spatial expression patterns of Dscam1. ***A***, Representative projection images of immunocytochemistry with α-GFP (green) to detect Dscam1^GFP^ and with the active zone marker α-brp (magenta) to label neuropil regions in *Drosophila* brain at different developmental stages of postembryonic life. ***B***, Dscam1^GFP^ expression through VNC development. ***C***, Images for all stages have been acquired with identical settings, Dscam1^GFP^ labeling intensities measured from projection views of VNC neuropils of three preparations per stage, normalized to maximal intensities at stage P7, and plotted over development. Time periods of motoneuron dendrite growth and synaptogenesis in VNC are indicated at the bottom. Dscam1 expression levels in VNC neuropils peak at pupal stages of fast motoneuron dendrite growth and synaptogenesis. L3 is larval stage 3, P3, P4, P5, P7, P10, P11, and P15 are pupal stages 3 to 15.

The descriptive anatomic analysis of spatiotemporal Dscam1 expression patterns during larval, pupal, and adult stages (Fig. 1) was confirmed in at least five biological replicates. For quantification of motoneuron structure (see Fig. 3) and neuromuscular physiology and structure (see Fig. 4) with Dscam1-kd compared with control, five or more different animals per genotype were analyzed. For dendritic structure analysis of Dscam1 mutant MARCM motoneurons at least three animals per mutant were analyzed. All quantification was conducted blindly without knowledge of the genotype. Statistical analysis was conducted with GraphPad Prism 8.3. Testing for effects between two experimental groups was performed with an unpaired, two-sided Student’s *t* test after confirming normal distribution with the Shapiro–Wilk test. For comparison of more than two experimental groups, a one-design ANOVA with Tukey’s test for pairwise *post hoc* comparison was used. Significance was accepted at *p < 0.05, **p < 0.01, ***p < 0.001. Comparison of dendrite structure for larval and adult aminergic and peptidergic neurons (see Fig. 5), as well as for different types of interneurons (see Figs. 6, 7) was qualitative. Biological replicates were from at least 10 different animals for each type of neuron. Qualitative structural differences (see Fig. 5) were consistent among all preparations. In case no qualitative structural differences are reported (see Figs. 6, 7), two different experts were unable to correctly predict the genotypes on random presentation of images from control animals and animals with targeted Dscam1-kd.

## Results

A fundamental prerequisite for the hypothesis that Dscam1 provides a code for arbor spacing in all *Drosophila* neurons is ubiquitous Dscam1 protein localization throughout the CNS during critical developmental stages of neuronal differentiation. Imaging Dscam1 protein with endogenous GFP tag (w; Dscam1^MiMICGFP^; Kamiyama et al., 2015) confirms abundant expression throughout all neuropils of the developing brain (Fig. 1*A*) and the developing VNC (Fig. 1*B*).

Although Dscam1 is detected in all CNS neuropils already at larval stage 3 (L3; Fig. 1) and L1 (see Fig. 3*L*), expression is markedly increased between pupal stage 3 (P3) and P11 (Fig. 1), developmental stages of dendritic and axonal growth (Ryglewski et al., 2014a). Dscam1 protein expression decreases in pharate adults, when dendritic and axonal growth cease and adult neural circuits have formed. Therefore, Dscam1 spatial and temporal protein expression patterns during postembryonic development are consistent with a ubiquitous role in neuronal arbor self-avoidance.

**Figure 2.**
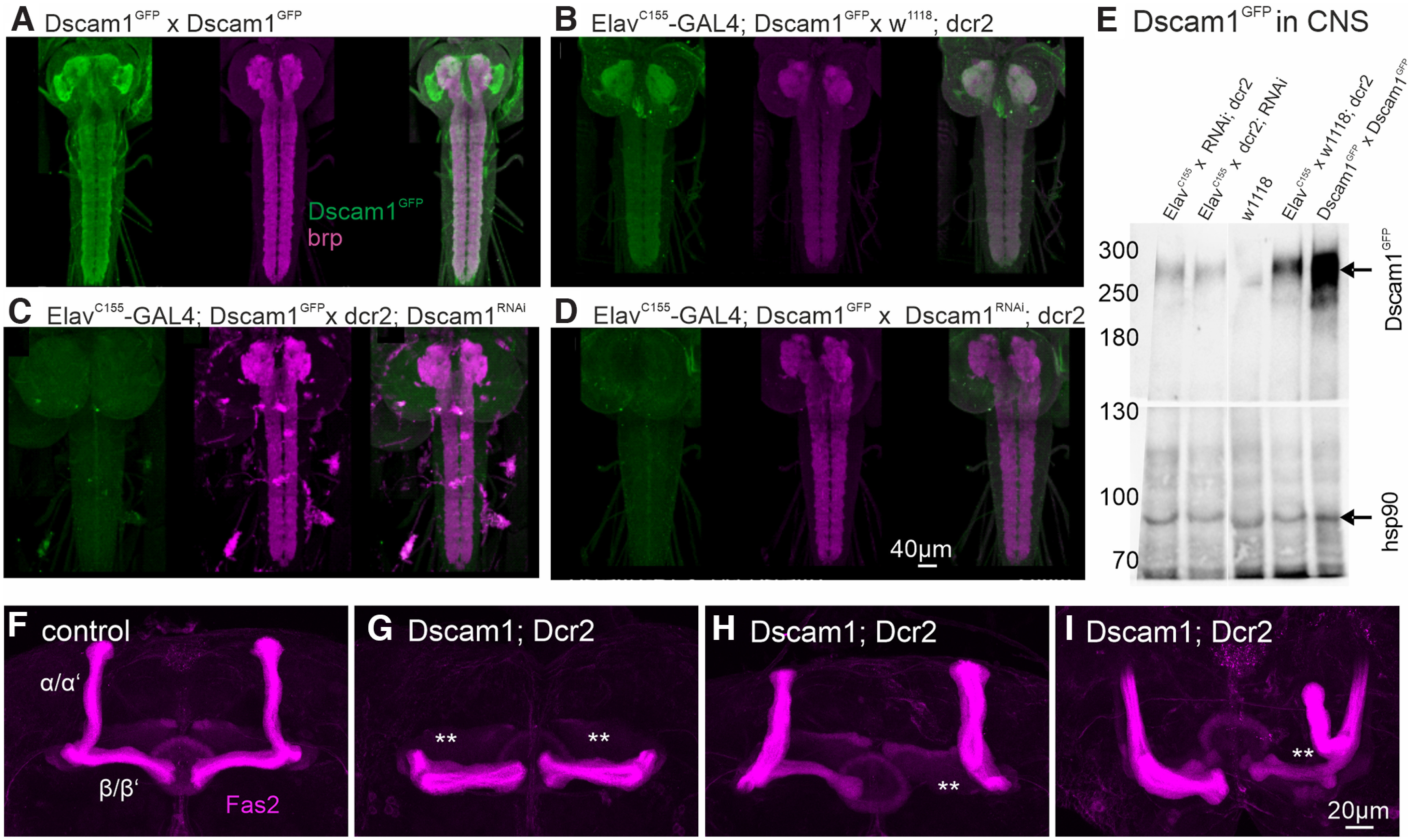
Dscam1-RNAi knocks down protein and reproduces known CNS phenotypes. ***A***, Colabeling of Dscam1^GFP^ (green) and synaptic neuropil (brp; magenta) in the larval CNS of homozygous Dscam1^GFP^ animals. ***B***, Heterozygous Dscam1^GFP^/Dscam1 animals show similar expression patterns but lower Dscam1^GFP^ labeling intensity (reduction by 42 ± 5%). ***C***, ***D***, Pan-neuronal RNAi knockdown of Dscam1 under the control of elav^C155^-GAL4 with two different UAS-RNAi transgenes (***C***; catalog #GD36233, VDRC; ***D***; catalog #KK108835, VDRC) reduced the Dscam1^GFP^ label below detection threshold (reduction by 90 ± 4% compared with control). ***E***, Western blotting for Dscam1^GFP^ and hsp90 (loading control) from adult *Drosophila* VNC homogenate. The Dscam1^GFP^ is detected at its expected size of ∼270 kDa in homozygous and heterozygous Dscam1^GFP^ animals but absent in controls. Both Dscam1 knockdowns reduce signal by ∼80%. ***F–I***, α-Fas2 immunolabel of adult MB in control (***F***) and following the expression of Dscam1-kd (catalog #KK108835, VDRC) in MB under the control of C739-GAL4 in three animals with representative kd phenotypes (***G–I***).

Compelling evidence for Dscam1-mediated dendritic self-avoidance exists for *Drosophila* dendritic arborization (da) neurons (Hughes et al., 2007; Matthews et al., 2007; Soba et al., 2007), but mechanosensory neuron dendrites are located in the larval body wall and do not receive synaptic input. With regard to central neurons that receive synaptic input to their dendrites, Dscam1 is reportedly required for dendrite spacing of adult olfactory interneurons (Zhu et al., 2006). In adult motoneurons, Dscam1 is required for normal dendritic arbor growth (Hutchinson et al., 2014). To test for the generality of Dscam1 function in central neuron dendrite development, we targeted Dscam1-RNAi kd selectively to different types of neurons with the binary UAS/GAL4 expression system. Although we have previously demonstrated RNAi-mediated Dscam1-kd in developing adult *Drosophila* motoneurons (Hutchinson et al., 2014), we examined the effectiveness of our Dscam1-kd by three means. First, pan-neuronal expression of Dscam1-kd under the control of Elav-GAL4^C155^ nearly eliminates Dscam1 protein in the CNS (Fig. 2*A–D*). The CNS of third instar larvae homozygous for Dscam1^GFP^ (Fig. 2*A*) shows brighter GFP fluorescence than heterozygous larvae (Dscam^GFP^/Dscam; Fig. 2*B*). Pan-neuronal expression of two different Dscam1-RNAi constructs nearly eliminates Dscam1^GFP^ signal (Fig. 2*C*,*D*). Second, these data are confirmed by Western blotting of adult brains (Fig. 2*E*). Dscam1^GFP^ yields a band at the expected size of 254 kDa that is weaker in heterozygous Dscam1^GFP^ animals, absent in control animals, and reduced by 85% with pan-neuronal Dscam1-kd (Fig. 2*E*). Third, targeting Dscam1-RNAi-kd to the developing MB recapitulates the phenotypes that have been reported on Dscam1 knockout (ko) and following critical reduction of Dscam1 isoform diversity (Hattori et al., 2007, 2009). These data confirm that our Dscam1 RNAi transgenes strongly reduce Dscam1 protein expression in *Drosophila* neurons and phenocopy the central brain developmental defects that provide compelling evidence for a role of Dscam1 in *Drosophila* central neuron arbor self-avoidance.

**Figure 3.**
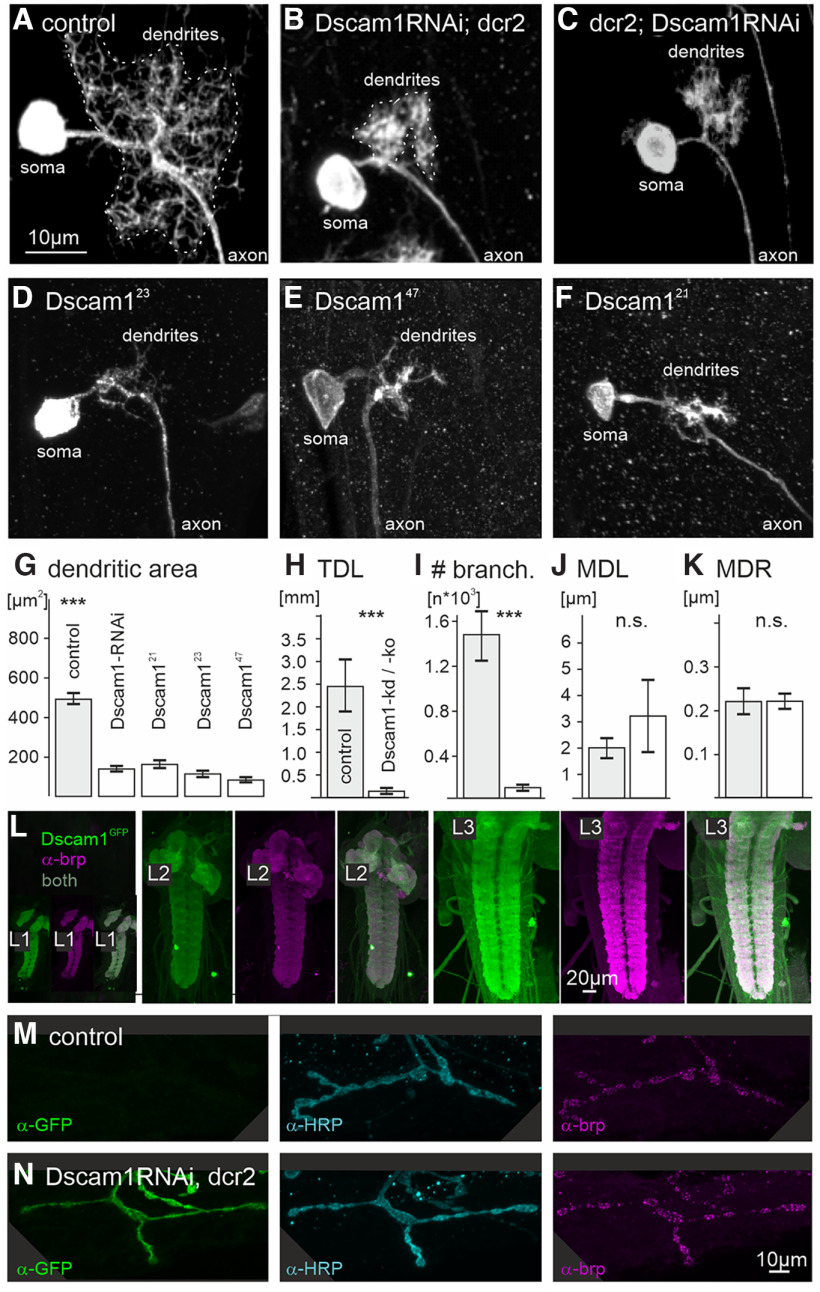
Dscam1 is required for larval MN dendrite but not axon differentiation. ***A–F***, Maximum intensity projection views of CLSM image stacks of GFP-labeled RP2 motoneurons in third instar larvae in control (***A***), with two different Dscam1-RNAi-knockdowns (***B***; catalog #KK108835, VDRC; ***C***, catalog #GD36233, VDRC), and with a Dscam1^23^ (***D***), a Dscam1^47^ (***E***), and a Dscam1^21^ (***F***) mutant allele. All mutant RP2 motoneurons were homozygous mutant in an otherwise heterozygous background with MARCM. ***G***, Dendritic coverage area as measured from projection views is significantly (***p < 0.001, ANOVA with Tukey’s *post hoc* comparison) reduced in all genotypes with reduced Dscam1. ***H–K***, Morphometric analysis reveals a significant reduction of TDL (***H***; ***p < 0.001, unpaired two-sided Student’s *t* test) and number of branches (***I***; **p < 0.01, unpaired two-sided Student’s *t* test**)** with reduced Dscam1, but the MDLs (***J***; n.s. unpaired two-sided Student’s *t* test) and mean dendritic radius (MDR; ***K***; n.s. unpaired two-sided Student’s *t* test) remain unaltered. ***L***, Dscam1^GFP^ expression (green) and active zone labeling with brp immunolabel (magenta) in the VNC during the first (L1, left), second (L2 middle), and third larval instar (L3, right) stages. Larval motoneuron total dendrite length is increased ∼10-fold between L1 and L3. ***M***, ***N***, Using a flippase strategy (see Materials and Methods), we created mosaic larvae with RP2 motoneurons that either express GFP and Dscam1-kd or not. α-HRP (cyan) and α-brp (magenta) labeling reveals no obvious differences in axon terminals and active zones between GFP-negative control (***M***) and GFP-positive Dscam1-kd RP2 cells (***N***).

We next tested the effects of Dscam1-kd and Dscam1 mutations on *Drosophila* larval RP2 motoneuron dendrite shape. Compared with control (Fig. 3*A*), with Dscam1-kd RP2 dendrites appear clumped and cover a much smaller neuropil area (Fig. 3*B*,*C*). Using MARCM (Lee and Luo, 2001; Kadas et al., 2017), we produced animals carrying GFP and three different Dscam1 mutations in subsets of larval RP2 motoneurons in an otherwise heterozygous background (see Materials and Methods). All three Dscam1 mutations (Fig. 3*D–F*) cause significant dendritic defects, similar to Dscam1-RNAi (Fig. 3*B*,*C*). Obtaining similar effects on dendrite structure for two different RNAi-kds and three different Dscam1 mutants renders unspecific effects of the manipulation unlikely. In all experiments, reduced Dscam1 function results in dendrites with a much smaller neuropil space coverage compared with the control (Fig. 3*A–G*). As reported for olfactory interneurons (Zhu et al., 2006), Dscam1-kd dendrites appear clumped, which is in accord with reduced dendritic self-avoidance and spacing. However, in larval motoneurons Dscam1-kd and Dscam1-ko also cause highly significant reductions in total dendrite length (Fig. 3*H*) and dendritic branch number (Fig. 3*I*). It was previously shown that Dscam1 is required for the initiation of dendritogenesis in the embryonic aCC motoneuron (Kamiyama et al., 2015). However, during embryonic life, aCC and its sibling RP2 motoneuron reach total dendrite lengths of <200 μm (Tripodi et al., 2008). By the third larval instar, RP2 total dendritic length (TDL) is ∼2500 μm (Fig. 3*H*), so that massive growth increases total dendrite length by a factor of ∼10 during larval life. We confirmed that Dscam1 is abundantly expressed in the VNC neuropils during these early larval stages of massive motoneuron dendrite growth and branching (Fig. 3*L*). Knockdown of Dscam1 in RP2 reduces total dendrite length at L3 by nearly 90% as previously reported for adult glutamatergic motoneurons (Hutchinson et al., 2014). By contrast, when Dscam1-mediated dendritic self-avoidance was first described in mechanosensory da neurons, Dscam1 mutant dendrites showed significantly increased overlaps with sister branches of the same neuron, but normal dendritic length and numbers of branch points (Matthews et al., 2007; Hughes et al., 2007; Soba et al., 2007). Therefore, the dendritic phenotypes of mechanosensory da neurons and motoneurons are different. In mechanosensory da neurons, loss of Dscam1 function causes exclusively increased self-contacts, whereas in motoneurons it causes increased self-contacts and reduced growth. It remains to be tested whether the primary cause for reduced dendritic length in motoneurons is a growth defect or a self-avoidance defect that impairs dendritic filopodial sprouting and consequently also dendrite growth (see Discussion).

**Figure 4.**
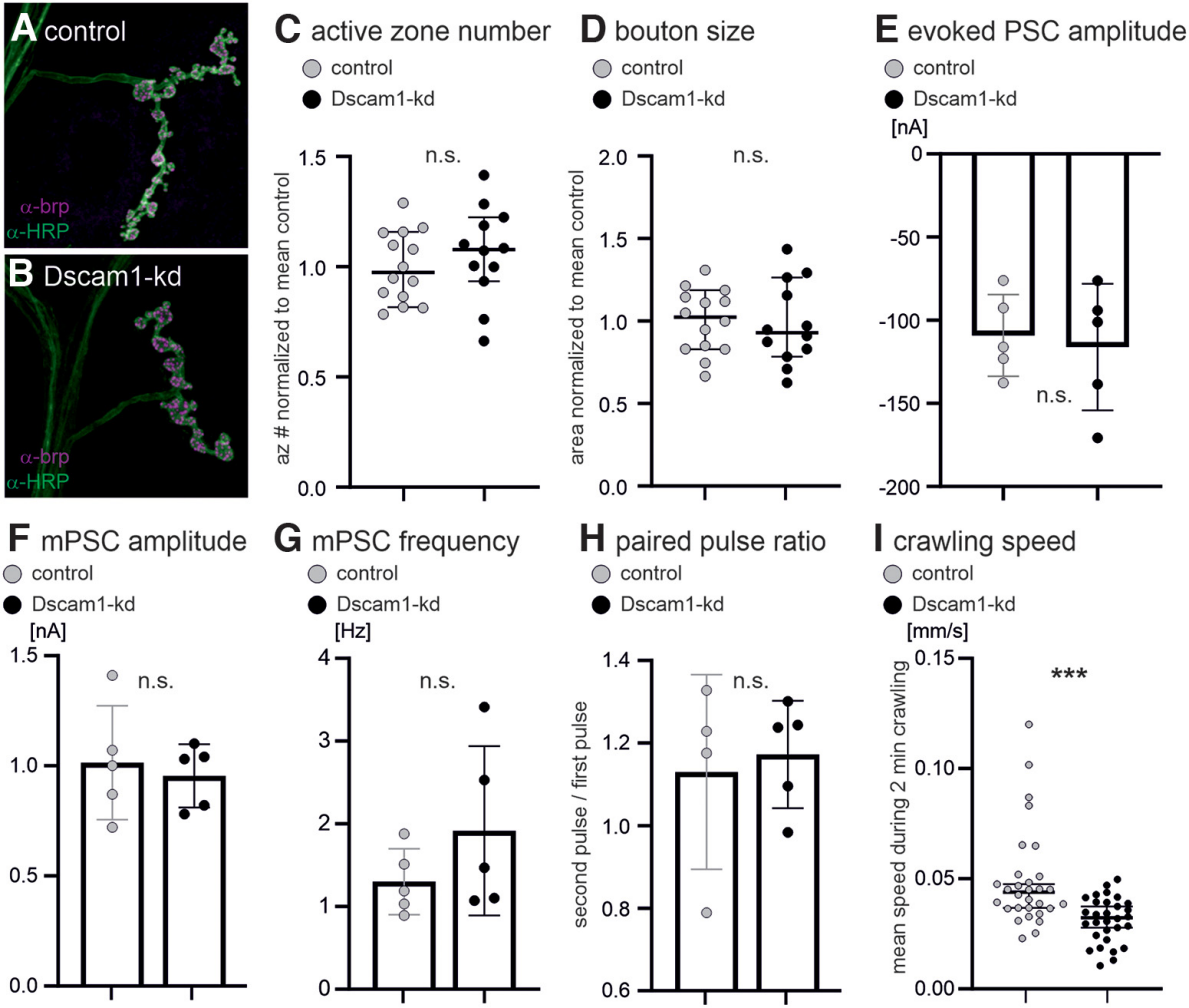
Dscam1 is not required for larval MN axon terminal differentiation. ***A***, ***B***, Representative motoneuron axon terminals on muscle 4 in control (***A***) and with Dscam1-kd (***B***). Quantification reveals no significant differences (n.s., unpaired two-sided Student’s *t* test) in active zone numbers (***C***), bouton size (***D***), evoked postsynaptic current (PSC) amplitude (***E***), miniature PSC amplitude (***F***) and frequency (***G***), or paired pulse ratio (***H***). ***F–H*** were recorded in two-electrode voltage-clamp mode. ***I***, Crawling speed was significantly reduced (***p < 0.001, unpaired, two-sided Student’s *t* test) in animals with Dscam1-kd in larval motoneurons.

By contrast, axon terminals and neuromuscular synaptic transmission are not affected by Dscam1-kd in motoneurons either in the larval stage (Figs. 3*M*,*N*, 4) or in the adult (Ryglewski et al., 2014b). To test for potential effects of Dscam1-kd on RP2 motoneuron axon terminal structure, we targeted GFP together with Dscam1-RNAi to subsets of RP2 motoneurons (flippase strategy, see methods). GFP-positive label serves as a marker for RP2 neurons with Dscam1-kd (Fig. 3*N*), all axon terminals are labeled with HRP (Fig. 3*M*,*N*), and presynapses are labeled with the presynaptic AZ marker brp (Fig. 3*M*,*N*). Upon Dscam1-kd no obvious differences are observed in RP2 axon terminal structure (Fig. 3*N*) compared with internal controls in adjacent segments of the same animals (Fig. 3*M*). Further quantification with Dscam1-kd in most larval crawling motoneurons under the control of the GAL4 driver D42 (Sanyal, 2009) reveals no differences in the number of presynaptic active zones (Fig. 4*A*,*B*), bouton size (Fig. 4*C*), the amplitude of evoked postsynaptic currents (Fig. 4*E*), miniature postsynaptic current amplitude or frequency (Fig. 4*F*,*G*), or short-term synaptic plasticity, as assayed by paired pulse ratio (Fig. 4*H*). However, crawling speed is slightly but significantly reduced on Dscam1-kd in larval crawling motoneurons (Fig. 4*I*). Given that axon terminal structure and neuromuscular transmission are not affected by Dscam1-kd in crawling motoneurons, reduced locomotion speed is likely a consequence of smaller dendrites and thus reduced synaptic input. Although we have not quantified the number of input synapses on larval crawling motoneurons with reduced dendrites, this interpretation is in agreement with previous observations of Dscam1-kd-mediated dendritic defects in adult motoneurons. Despite significant reductions in dendrite length and neuropil area coverage, some presynaptic partners are still targeted correctly to motoneuron dendrites, so that basic locomotor tasks can be performed with highly reduced motoneuron dendrites. However, smaller dendritic fields receive fewer synaptic inputs so that fine motor skills or fast locomotor performance are impaired (Ryglewski et al., 2014b).

In sum, Dscam1-kd does not affect axonal growth and presynaptic function, but it severely impairs dendrite growth in larval and adult *Drosophila* glutamatergic motoneurons. With reduced Dscam1 function, motoneuron total dendritic length and dendritic branch numbers are significantly reduced, and the remaining dendrites appear clumped and cover a significantly reduced synaptic input space. We next assayed two additional types of efferent neurons with dendrites in the VNC and axonal projections in segmental nerves.

**Figure 5.**
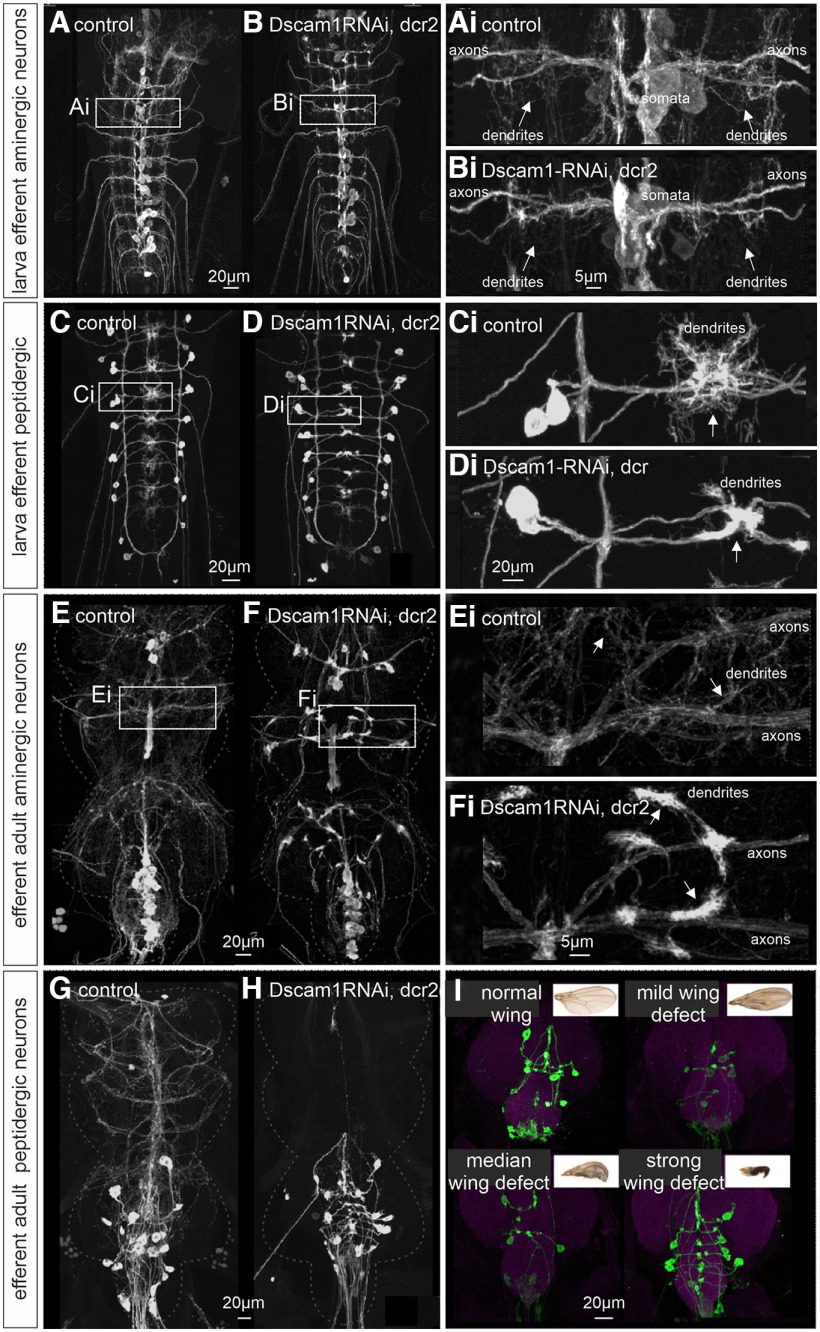
Dscam1 is required for normal dendrites in aminergic and peptidergic neurons. ***A***, ***B***, Projection views of ventral unpaired median aminergic neurons in the larval VNC that are labeled with UAS-GFP expressed under the control of TDC2-GAL4. White boxes in control (***A***) and with Dscam1-kd (***B***) are selectively enlarged in ***Ai*** and ***Bi***. Dscam1-kd eliminates higher-order dendritic branches (see white arrows). ***C***, ***D***, Projection views of larval peptidergic neurons with UAS-GFP expression under the control of CCAP-GAL4. White boxes in control (***C***) and with Dscam1-kd (***D***) are selectively enlarged in ***Ci*** and ***Di***. Dscam1-kd eliminates higher-order dendritic branches and causes dendrite clumping (see white arrows). ***E***, ***F***, Projection views of ventral unpaired median aminergic neurons in the adult VNC that are labeled with UAS-GFP expressed under the control of TDC2-GAL4. White boxes in control (***E***) and with Dscam1-kd (***F***) are selectively enlarged in ***Ei*** and ***Fi***. Dscam1-kd causes dendrite clumping (see white arrows). ***G***, ***H***, Projection views of adult peptidergic neurons with UAS-GFP expression under the control of CCAP-GAL4. Dscam1-kd reduced dendritic branches in VNC abdominal segments and nearly eliminates CCAP neuron dendrites from the thoracic VNC neuromeres. ***I***, Dscam1-kd in adult CCAP neurons goes along with wing defects. Both CCAP neuron morphologic defects and wing defects show variable severity, but there is no correlation between the severity of neuronal and wing phenotypes.

In addition to the type 1 axon terminals of excitatory glutamatergic motoneurons, many *Drosophila* muscles are innervated by type II terminals of aminergic modulatory neurons (Atwood et al., 1993; Pflüger and Duch, 2011; Stocker et al., 2017) and type III terminals of peptidergic neurons (Anderson et al., 1988; Dulcis and Levine, 2003). To probe the role of Dscam1 also in aminergic and peptidergic efferent neurons, we expressed Dscam1-kd selectively under the control of TDC2-Gal4 [TDC2 (tyrosine decarboxylase)] in octopaminergic neurons (Stocker et al., 2017) and under the control of CCAP-GAL4 (CCAP) in CCAP-releasing neurons (Park et al., 2003). In both the larval stage (Fig. 5*A–D*) and the adult stage (Fig. 5*E–I*), Dscam1-kd causes dendritic defects in these types of neurons. TDC2-GAL4 drives expression in unpaired octopaminergic neurons that are located in each segment along the ventral midline of the VNC. In the larval stage (Fig. 5*A*, control, *B*, Dscam1-kd), Dscam1-kd has no obvious effects on the location of the somata or the axonal projections into different segmental nerves, but it eliminated higher-order dendritic branches (Fig. 5*Ai*,*Bi*, arrows). This is indicative of a dendritic growth defect, because the remaining dendritic branches show no clumping. CCAP-GAL4 drives expression in peptidergic neurons with lateral cell bodies on both sides of each segment of the VNC, axonal projections into the segmental nerves, and dendrites that branch along the VNC midline (Fig. 5*C*, control, *D*, Dscam1-kd). In the larval stage Dscam1-kd has no obvious effects on CCAP neuron somata or axonal projections into segmental nerves, but it causes clumped dendrites with a smaller neuropil area coverage compared with control (Fig. 5*Ci*,*Di*, arrows). A similar dendritic phenotype is observed for Dscam1-kd in adult octopaminergic neurons (Fig. 5*E*,*F*). Here spaced higher-order dendritic branches that are present in controls (Fig. 5*E*,*Ei*, arrows) are absent with Dscam1-kd (Fig. 5F) and replaced by bright dendritic clumps (Fig. 5*Fi*, arrows). Axonal bifurcations and projections into the correct segmental nerves (Fig. 5*E*,*F*) as well as the axon terminal structure on the muscle (data not shown) show no obvious structural differences with control. A severe phenotype is observed with Dscam1-kd in adult CCAP neurons (Fig. 5*G*,*H*). CCAP neuron arborizations in the CNS are reduced in the abdominal segments but are mostly absent from all thoracic segments. The nearly complete absence of CCAP neuron dendritic branches in thoracic neuromeres is indicative of a dendritic growth defect with Dscam1-kd. Although CCAP neuron central arbors are always impaired on Dscam1-kd, we observe animal-to-animal variation in phenotype severity (Fig. 5*J*). Phenotypic variation unlikely results from variable GAL4 expression, because the CCAP-GAL4 expression is highly reproducible between animals. Variable dendritic phenotypes on GAL4-driven Dscam1-RNAi have also been reported for adult flight motoneurons (Hutchinson et al., 2014). Dendritic phenotypes, as observed with Dscam1-kd under the control of CCAP-GAL4, go along with variable defects in wing morphology. Since CCAP is involved in the peptidergic control of molting, wing phenotypes may result from aberrant ecdysis patterns. However, the severity of the wing phenotypes observed does not seem to correlate with the severity of the dendritic phenotypes (Fig. 5*I*). Dendritic defects as observed in octopaminergic neurons did not cause any obvious viability or locomotor defects. This is no surprise, because the ablation of TDC2 neurons causes only quantitative locomotor performance impairments (Brembs et al., 2007; Schützler et al., 2019).

In sum, in all three types of *Drosophila* neurons with efferent axonal projections and branched dendrites in the CNS, Dscam1 is not required for axonal growth and target finding, but reduced Dscam1 function severely impairs dendritic architecture. In each case, Dscam1-kd severely reduces normal dendrite length and branch numbers. In larval (Fig. 3) and adult (Hutchinson et al., 2014) motoneurons, adult octopaminergic neurons (Fig. 5*E*,*F*), and larval CCAP neurons (Fig. 5*B*,*C*), the remaining dendrites appear clumped. As mentioned above, dendrite clumping is indicative of impaired self-avoidance with Dscam1-kd. Whether reduced dendrite length and branch numbers result from an additional growth-related function of Dscam1 in these neuron types, or whether impaired self-avoidance during dendritic filopodia movement results in growth defects remains to be investigated (see Discussion). However, the dendritic phenotypes that we observe with Dscam1-kd in larval octopaminergic neurons (Fig. 5*A*,*B*) and in adult CCAP neurons (Fig. 5*G*,*H*) indicate primary dendritic growth defects. Here, the lack of higher-order dendrites (larval TDC2 neurons) or the loss of nearly all dendritic branches is not accompanied by clumping of the remaining dendrites. Therefore, our data on different types of efferent neurons support a role of Dscam1 in dendrite spacing, but indicate additional functions of Dscam1 on dendrite growth and branching. Interestingly, even the same neuron type can show combined self-avoidance (dendrite clumping) and growth (shorter dendrite length) in one stage (e.g., adult TDC2 neurons), but show growth defects without clumping in another developmental stage (e.g., larval TDC2 neurons). This indicates that Dscam1 may have diverse effects on dendritic differentiation that are neuron type and developmental stage specific. To further probe this possibility, we next tested the role of Dscam1 on dendritic shape development in different types of interneurons in the VNC and the brain.

**Figure 6.**
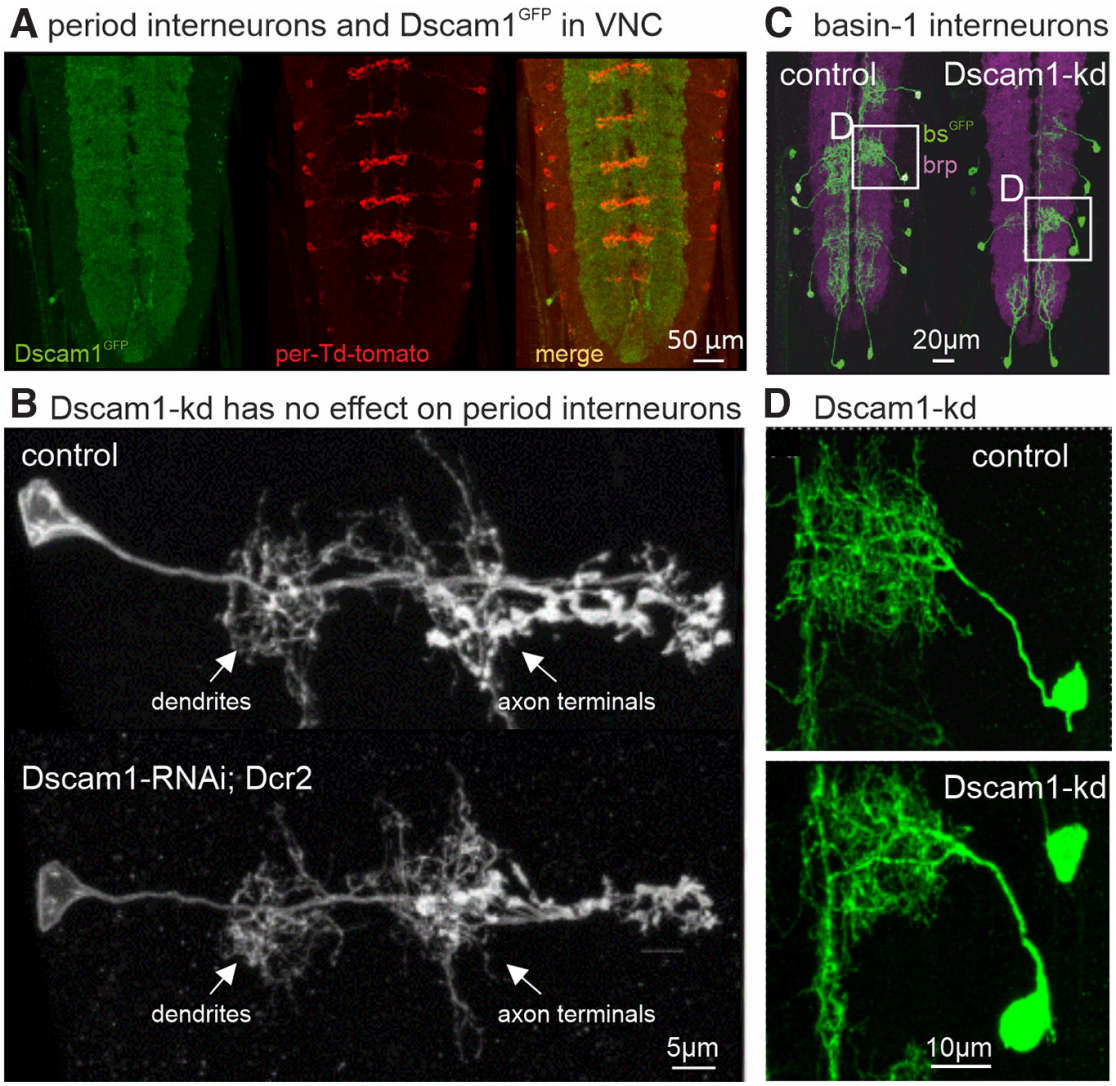
Dscam1 is not required for VNC interneuron arbor differentiation. ***A***, Expression of tdTomato under the control of per-GAL4 in animals with GFP-tagged endogenous Dscam1 reveals that all axonal and dendritic arbors of PMSIs (red) are located Dscam1^GFP^ (green)-positive VNC neuropil areas. ***B***, Dscam1-kd in PMSIs has no obvious effects on the axonal or dendritic structure of these interneurons. ***C***, Mosaic labeling of basin-1 interneurons (green) in larval VNC counterstained with the synaptic marker brp (magenta) in control and with Dscam1-kd in basin-1 neurons. ***D***, Selective enlargement reveals no differences between control and Dscam1-kd basin-1 interneurons.

For the analysis of Dscam1 function in interneuron dendrite development, we chose interneurons of different transmitter classes for which selective GAL4 driver lines are available. First, we used period-GAL4 to drive expression of reporters and/or Dscam1-kd in glutamatergic, period-positive, median segmental interneurons (PMSIs), which regulate the speed of *Drosophila* larval crawling (Kohsaka et al., 2014; Itakura et al., 2015). Expression of UAS-tdTomato under the control of per-Gal4 in a Dscam1^GFP^ background reveals that all branched processes (axons and dendrites) of PMSIs are surrounded by neuropil with abundant Dscam1 expression (Fig. 6*A*). Targeted expression of Dscam1-kd in PMSIs (Fig. 6*B*, top) has no obvious effect on dendritic or axonal structure (Fig. 6*B*, bottom). In control and with Dscam1-kd, PMSI dendrites occupy similarly sized neuropil areas and are of akin shape with regard to branch numbers, length, and spacing (Fig. 6*B*). Thus, Dscam1 is unlikely to be required for arbor differentiation of PMSIs. We next examined the role of Dscam1 on the development of basin-1 interneurons. Basin-1 is one of four segmentally repeated types of projection neurons (basin-1 to 4) with basin-shaped arbors in the VNC that receive input in response to vibrational activation of chordotonal organs (Itakura et al., 2015; Ohyama et al., 2015). We used mosaic labeling of basin-1 neurons in control and with Dscam1-kd (Fig. 6*C*). Selective enlargements of basin-1 interneuron dendrites reveal no qualitative structural differences between control and Dscam1-kd (Fig. 6*D*). Dendrites show similar lengths, branch numbers, neuropil coverage, and spacing (Fig. 6*D*). Although our analysis of the structure of PMSI and basin-1 interneurons remains qualitative, we can exclude major growth or self-avoidance defects. However, we cannot exclude more intricate structural differences between Dscam1-kd and control in these neuron types (this also applies for brain interneurons; see below).

**Figure 7.**
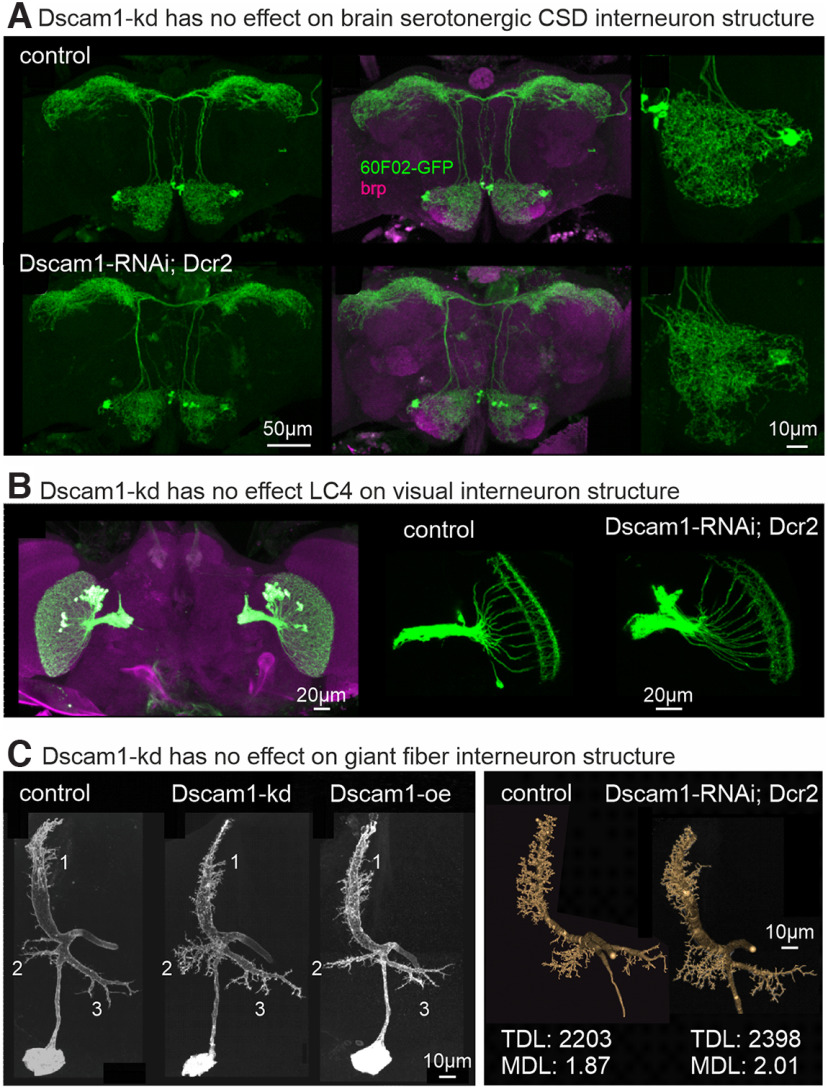
Dscam1 is not required for brain interneuron arbor differentiation. ***A***, Representative projection view of GFP expression in serotonergic CSD interneurons (green) in the adult *Drosophila* brain counterstained with the synaptic marker brp (magenta, middle). CSD interneuron structure shows no obvious differences between control (top left, top middle) and Dscam1-kd (bottom left, bottom middle). Selective enlargements of CSD neuron arbors in antennal lobe (right) reveals no obvious differences between control (top right) and Dscam1-kd (bottom right). ***B***, Overview of LC4 neuron structure in the adult brain counterstained with brp (left). Dscam1-kd (right) has no obvious effect on LC4 neuron arbor lengths or spacing compared with control (middle). ***C***, Left, Representative projection views of GF interneuron dendrite structure in control (left), with Dscam1-kd (middle), and following Dscam1 overexpression. All three dendritic subtrees (numbered) contain higher-order dendrites, which are normally spaced relative to each other. Right, Representative quantitative reconstructions of control and Dscam1-kd GF interneurons with similar total dendrite length and mean dendritic branch length.

So far, the only neuron type in the *Drosophila* CNS for which a dendritic self-avoidance phenotype has been reported is olfactory interneurons in the *Drosophila* brain (Zhu et al., 2006). We tested three additional types of brain interneurons for the requirement of Dscam1 in dendritic self-avoidance and spacing. First, we used 60F02-GAL4 to drive UAS-Dscam1-kd in contralateral serotonergic deuterocerebral (CSD) interneurons. CSD interneurons make output synapses to and receive input synapses from multiple sensory networks in the *Drosophila* brain (Coates et al., 2020). Compared with control, targeted expression of Dscam1-kd in CSD interneurons reveals no differences in the arborization patterns in any of the brain neuropils (Fig. 7*A*), either in whole-brain overview images (Fig. 7*A*, left, middle) or in selective enlargements of CSD arbors in antennal lobe (Fig. 7*A*, right). These data suggest that Dscam1-kd has no severe effects of CSD interneuron central arbor structure.

Similar observations were made for LC4 interneurons, a population of visual interneurons that receive synaptic input into their dendrites in the medulla. Targeting Dscam1-kd to LC4 interneurons revealed no obvious differences in dendritic length, branching, coverage, or spacing compared with control (Fig. 7*B*). In addition, we tested whether normal dendrite structure of the GF (Koto et al., 1981) descending interneuron requires Dscam1. The GF receives visual and mechanosensory synaptic inputs to its dendrites in the brain. We used a split-GAL4 line that drives expression of UAS-transgenes selectively in the GF neurons (von Reyn et al., 2014). GF dendrite structure appears similar in control, with Dscam1-kd, and with overexpression of one Dscam1 isoform (Fig. 7*C*). In each genotype, the three major dendritic subtrees are present and oriented in the same angles relative to each other (Fig. 7*C*, left panels). Quantitative 3-D reconstructions (Evers et al., 2005) of representative preparations reveal no marked differences in TDL, the number of branches, or the mean length of dendritic branches (MDLs) between Dscam1-kd and control (Fig. 7*C*, right panels). In sum, none of the three different types of brain interneurons show any dendritic defects on Dscam1-kd.

## Discussion

To test whether Dscam1 is used for neuronal arbor self-avoidance in all *Drosophila* neurons, or whether Dscam1 has diverse neuron-type and developmental stage-specific functions, we targeted Dscam1-kd at different developmental stages to different types of neurons in the *Drosophila* CNS. Dscam1 spatial and temporal expression patterns in all brain and VNC neuropils at all critical stages of postembryonic neuronal arbor differentiation support the possibility that Dscam1 could affect the differentiation of all neurons. In fact, Dscam1 is required for normal dendritic architecture development in the three different types of efferent neurons that we tested, both in the larval and the adult stage. However, in efferent neurons Dscam1-kd affects dendritic arbor spacing and growth to different degrees, depending on neuron type and developmental stage (see below; Table 2). By contrast, the five different types of interneurons that we tested in this study do not require Dscam1 for normal dendrite development (see below; Table 2). Axonal arbor development is not affected in any of the efferent neurons or interneuron types assayed in this study, but clearly in some previously tested neuron types (He et al., 2014), and we have reproduced the MB phenotypes that result from aberrant self-avoidance of the axon arbors of MB principle neurons (Hattori et al., 2007). We conclude that Dscam1 is not required for arbor spacing in all *Drosophila* neurons, but instead, has differential and diverse roles during neuronal arbor development that depend on neuron type and developmental stage.

**Table 2:** Summary of the effects of Dscam1-kd on dendrites and axons of the different neuron types analyzed

Neuron type	Dendritic self-avoidance	Dendritic growth	Axonal self-avoidance	Axonal growth	Figures
Larval RP2 motoneurons	Some clumping	Strong defect	None	None	3, 4
Larval TDC2 aminergic neurons	Some clumping	Defect	None	None	5
Adult TDC2 aminergic neurons	Strong clumping	Defect	None	None	5
Larval CCAP peptidergic neurons	Strong clumping	Defect	None	None	5
Adult CCAP peptidergic neurons	*	Strong defect	Not assessed	Not assessed	5
Period interneurons in VNC	None	none	none	none	6
Basin interneurons in VNC	None	none	none	none	6
CSD brain interneurons	None	none	none	none	7
LC4 visual interneurons	None	none	none	none	7
Giant fiber interneurons	None	none	none	none	7

*Not analyzed because of strong growth defect.

### Are dendritic defects in efferent neurons with Dscam1-kd caused by self-avoidance or dendritic growth impairments?

In larval and adult excitatory glutamatergic motoneurons with type I boutons at the neuromuscular junction (NMJ), adult modulatory aminergic neurons with type II boutons at the NMJ, and larval peptidergic neurons, Dscam1-kd causes dendrite clumping. Note that this is not observed in larval aminergic and adult peptidergic neurons, indicating that the same type of efferent neuron is differentially affected by Dscam1-kd at different developmental stages. Dendrite clumping has previously been reported for olfactory interneuron dendrites and is indicative of defective self-avoidance (Zhu et al., 2006). However, in addition to developmental stage and neuron type-specific dendrite clumping, at all stages and in all types of efferent neurons investigated, Dscam1-kd also causes marked reductions in the number of dendritic branches and in total dendritic length, which may indicate additional growth defects. The mechanisms that underlie decreased dendrite growth in these neurons with Dscam1-kd remain elusive, but it is possible that dendritic growth and branching are mechanistically linked to self-avoidance. Given that Dscam1 isoform diversity provides each neuron with a unique surface identity (Neves et al., 2004) and that only interactions between identical Dscam1 isoforms promote contact-dependent repulsion (Wang et al., 2002; Wojtowicz et al., 2004), it is conceivable that repulsion between newly formed sister branches promotes dendritic bifurcation and subsequently dendritic field coverage. Accordingly, in olfactory interneurons, Dscam1-ko causes a smaller dendritic area coverage, whereas Dscam1 overexpression results in more diffuse dendritic arbors (Zhu et al., 2006). Therefore, it is possible that the primary cause for reduced dendrite lengths in efferent neurons with Dscam1-kd is impaired self-avoidance. However, three observations are not consistent with this idea. First, in larval aminergic neurons Dscam1-kd causes reduced dendrite length and branch numbers without dendrite clumping. Second, in adult *Drosophila* motoneurons Dscam1-kd or -ko impairs the formation of new dendritic branches already during early stages of dendritic growth (Hutchinson et al., 2014). Third, in adult CCAP neurons, most dendrites are not formed. These observations indicate a primary growth defect instead of a dendritic self-repulsion defect on Dscam1-kd. However, we cannot exclude the possibility that nascent dendritic filopodia require Dscam1-mediated self-repulsion to form new dendritic branches. For adult motoneuron growth, the idea that Dscam1 may interact with signaling pathways that regulate dendritic filopodia and growth-cone dynamics has been suggested (Hutchinson et al., 2014). Normal filopodia dynamics may require Dscam1-mediated self-repulsion, so that new dendritic branches are not formed with impaired filopodial self-avoidance. In fact, during mechanosensory axon collateral formation in the *Drosophila* VNC, Dscam1 signaling is required for normal growth-cone sprouting (He et al., 2014). There, multiple isoforms are required cell-intrinsically for normal arbor growth, and it has been suggested that the ratio of matching and nonmatching Dscam1 isoforms within a single neuron can affect growth-cone sprouting (He et al., 2014). Although our RNAi-mediated Dscam1-kd may also alter relative isoform abundance, we judge cell intrinsic isoform diversity an unlikely requirement for normal efferent neuron dendrite growth, because Dscam1 mutant motoneurons show highly similar dendritic phenotypes to Dscam1-kd motoneurons, both in the larval (this study) and in the adult stage (Hutchinson et al., 2014). Although it remains unclear through which mechanism Dscam1 supports dendritic growth and branching in different types of efferent *Drosophila* neurons, our data indicate that Dscam1 differentially affects efferent neuron dendrite differentiation depending on developmental stage and neuron type. Finally, it is noteworthy that axonal projections and presynaptic structures are not affected by Dscam1-kd in these types of neurons. By contrast, axonal phenotypes have previously been shown in sensory neuron projections in larval VNC (He et al., 2014; Dascenco et al., 2015) and in mushroom body principle neuron axon collateral segregation (Hattori et al., 2007, 2009). Although we could recapitulate MB phenotypes by targeted expression of Dscam1-RNAi, the same RNAi transgenes did not affect axon arbors of different types of efferent neurons. The mechanisms that mediate differential effects in different compartments of different types of neurons currently remain unknown.

### Arbor structure of multiple interneuron types does not require Dscam1

Neither the dendritic nor the axonal structure is affected by Dscam1-kd in any of the five different interneuron types that we have tested. Although Dscam1 isoform diversity, homophilic repulsion, as well as spatial and temporal expression patterns can provide a ubiquitous code for self-recognition for all *Drosophila* neurons, our data indicate that this code is not used for arbor spacing in multiple different types of neurons. Despite the broad expression of Dscam1 in nearly all neuropils and neuronal cell bodies throughout postembryonic development, we have not rigorously studied the expression levels of Dscam1 specifically in the identified interneuron types under investigation. We can thus not exclude the possibility that Dscam1 RNAi causes no structural phenotypes in these neurons because they simply do not express Dsacm1 at critical stages of arbor differentiation. However, either way the data suggest that Dscam1 is not used for dendritic self-avoidance in the identified interneurons assayed in this study.

Dscam1-mediated dendrite spacing has first been described for mechanosensory da neurons in the larval *Drosophila* body wall (Hughes et al., 2007; Soba et al., 2007; Matthews et al., 2007), but these neurons do not receive synaptic input to their dendrites. In the CNS, the requirement of Dscam1 for orderly axonal sprouting or bifurcation has been demonstrated in seminal studies on mechanosensory axonal branching in the VNC (He et al., 2014; Dascenco et al., 2015) and on the segregation of axon collaterals during MB development in the brain (Wang et al., 2002, 2004; Zhan et al., 2004; Hattori et al., 2007, 2009; Goyal et al., 2019). For dendrites that receive input synapses in the CNS, an instructive role of Dscam1 for correct arbor spacing has been shown only for olfactory interneurons (Zhu et al., 2006), but it remains unclear whether these neurons maintain normal dendrite lengths and branch numbers on loss of Dscam1 function. Therefore, Dscam1 might quite as well be required for self-avoidance and normal dendritic growth and branching of these neurons, as we find in this study for some efferent neurons. However, given that we add five additional types of interneurons in the CNS, and none requires Dscam1 for the development of normal dendrite architecture, we conclude that Dscam1 displays neuron type-specific effects on different aspects of axonal and dendritic differentiation. With regard to an exclusive function for dendrite spacing, Dscam1 is clearly required in mechanosensory da neurons, but not in many central neurons.

At present, we can only speculate about the mechanisms that underlie dendritic arbor spacing in interneurons that do not rely on Dscam1 signaling. In contrast to da neurons, all central neurons receive synaptic input to their dendrites. A possible alternative mechanism for central neuron dendrite spacing might be synaptotropic growth, a two-step process during which dendritic filopodia are first transformed into stable dendrites at sites of new synapse formation, which is followed by the formation of additional dendritic filopodia (Niell et al., 2004; Hassan and Hiesinger, 2015). Therefore, at any point in the developing neuropil space, only one new dendritic branch can be stabilized, and those without nascent synapses will retract. If potential presynaptic partners exist at minimal distances to each other, or arrive in temporal sequences at different neuropil locations, synaptotropic growth could mediate dendritic arbor spacing in the CNS. Synaptotropic growth has been demonstrated during the differentiation of *Drosophila* motoneuron dendrites (Ryglewski et al., 2017), but its role in central neuron dendrite spacing remains to be investigated. The relative contributions of Dscam1-mediated self-avoidance, Dscam1 interactions with other molecules, synaptotropic growth, or combinations of these to the correct spacing of central neuron arbors remain to be investigated.

## Synthesis

Reviewing Editor: Jeffery Twiss, University of South Carolina Decisions are customarily a result of the Reviewing Editor and the peer reviewers coming together and discussing their recommendations until a consensus is reached. When revisions are invited, a fact-based synthesis statement explaining their decision and outlining what is needed to prepare a revision will be listed below. The following reviewer(s) agreed to reveal their identity: NONE. Note: If this manuscript was transferred from JNeurosci and a decision was made to accept the manuscript without peer review, a brief statement to this effect will instead be what is listed below.

This manuscript was reviewed at JNS and transferred to eNeuro. Reviews are comprehensive and authors have provided a robust and I think complete response to their comments. The new data added strengthen the manuscript. Overall, I think that the authors have more than sufficiently addressed all of the reviewers points and this will be a nice addition to eNeuro. So I think the manuscript is in general appropriate for acceptance at eNeuro. The authors use the term ‘developing adult neuron’ in the introduction as well as in parts of the manuscript. I recognize that this is likely standard language for drosophila but I think the general eNeuro readership may be confused to see ‘developing’ used to describe an adult neuron. I suggest that the authors provide 1-2 sentences to clarify this term when first used and the uniqueness of their model system so that more mammalian inclined readership will not be misunderstand the author’s interpretations.
